# Exosome-Mediated Transfer of miR-3613-5p Enhances Doxorubicin Resistance by Suppression of PTEN Expression in Breast Cancer Cells

**DOI:** 10.1155/2022/9494910

**Published:** 2022-10-14

**Authors:** Lin Luo, Xin Zhang, Yiliyaer Rousuli, Alibiyati Aini

**Affiliations:** ^1^Department of Head and Neck Surgery, Affiliated Cancer Hospital of Xinjiang Medical University, Urumqi, Xinjiang Uygur Autonomous Region 830011, China; ^2^Department of Medical Administration, Affiliated Cancer Hospital of Xinjiang Medical University, Urumqi, Xinjiang Uygur Autonomous Region 830011, China

## Abstract

Breast cancer is the most common malignancy among women worldwide, and patients easily develop resistance to the first-line drug doxorubicin. To elucidate the molecular mechanism of drug resistance in breast cancer is imperative. Exosomes mediate the crosstalk between neighboring cells and intercellular communication. Incorporation of miRNAs into exosomes prevents the degradation and facilitates the intercellular communication, which has been indicated in regulation of drug resistance. qRT-PCR revealed that miR-3613-5p is upregulated in drug-resistant breast cancer, and miR-3613-5p exists in exosomes. It is predicted that miR-3613-5p can bind to the tumor suppressor gene PTEN. In this study, our results showed that miR-3613-5p was upregulated in drug-resistant tissue and in exosomes of breast cancer cells resistant to doxorubicin. CCK8, crystal violet staining, and flow cytometry analysis demonstrated that exosome mediated miR-3613-5p transfer and enhanced the resistance to doxorubicin of breast cancer cells. Western blotting showed that miR-3613-5p could target PTEN and regulate the expression of PTEN. Exosome-mediated transfer of miR-3613-5p enhanced the resistance to doxorubicin by inhibition of PTEN in breast cancer cells.

## 1. Introduction

Breast cancer is the most common malignancy in women worldwide, and the incidence continues to rise [1]. Despite substantial progress and improvements have been achieved over past few decades, it is still a major cause of mortality [1]. Metastasis remains a leading cause of mortality in breast cancer patients, accounting for more than 90% of mortality [1, 2]. Doxorubicin is the most extensively used first-line drug for breast cancer treatment. However, the rapid development of drug resistance has fundamentally weakened its anticancer efficacy [3]. Therefore, it is imperative to explore the potential molecular mechanisms of doxorubicin resistance and find new therapeutic targets for breast cancer.

Emerging studies have demonstrated that exosomes secreted by cells can alleviate drug resistance and improve prognosis of malignancies [4–6]. Exosomes are nanoscale membrane vesicles with a diameter of 30-150 nm, and they participate in intercellular communication by transporting of lipids and nucleic acids to recipient cells [7]. Cell-secreted exosomes mediate the crosstalk between neighboring cells and transport to distal tissues, where signals and messages were sent to specific recipient cells [7].

MicroRNAs (miRNAs) are small noncoding RNAs with a length of about 22 nucleotides, which can posttranscriptionally regulate gene expression [8, 9]. Dysregulated miRNAs have been implicated in many different pathophysiological processes [10]. Multiple evidence indicate that miRNAs are involved in the regulation of drug resistance. miRNAs are protected by bilateral membrane structures upon its incorporation into exosomes, thereby reducing miRNA degradation and promoting intercellular communication [11]. Overexpressed miR-567 can be packaged into exosomes and incorporated into recipient cells, which then inhibits autophagy and reverses chemoresistance by targeting ATG5 [12]. miR-155 is induced in exosomes isolated from cancer stem cells and resistant breast cancer cells, and exosome-mediated transfer of miR-155 into breast cancer cells enhances resistance to chemotherapeutic drugs [13].

Based on the GEO database, miR-3613-5p is found to be upregulated in chemoresistant breast cancer, indicating that miR-3613-5p may be involved in the drug resistance of breast cancer. However, exosome-mediated miR-3613-5p transfer in drug resistance of breast cancer has not been studied yet. This present work demonstrated that exosome-mediated transfer of miR-3613-5p enhanced the resistance of breast cancer cells to doxorubicin by inhibition of PTEN.

## 2. Materials and Methods

### 2.1. Human Cell Lines and Reagents

MDA-MB-231 and MCF-7 cells were purchased from ATCC and maintained in rich DMEM. Fetal bovine serum was purchased from Thermo Fisher Scientific. CCK8 kit (96992) was purchase from Sigma-Aldrich.

### 2.2. RNase A Treatment

The culture medium was supplemented with 10 mg/ml RNase A and incubated at 37°C for 1 h to remove RNA contamination.

### 2.3. GEO Data Analysis

Gene Expression Omnibus (GEO) series dataset (GSE73736) was downloaded from GEO. Differential expression analysis in drug-resistant and sensitive tissue of breast cancer was conducted. Estimation of the relative subsets of RNA transcript was performed.

### 2.4. Transmission Electron Microscopy (TEM)

Exosomes solution was dropped onto the formvar grid. Filter paper was used to remove excess water. The exosomes were fixed with 2% phosphotungstic acid for 10 min and then rinsed with deionized water. Then, exosomes were stained with 1% uranyl acetate for 15 min. Philips EM208S TEM (Netherlands) at 100 kV was used to photograph the exosome's morphology.

### 2.5. Real-Time Quantitative PCR (qRT-PCR)

TRIzol (Invitrogen) was used for total RNA extraction. The reaction mixture was prepared according to the instruction of SYBR Green (Takara, Japan). The reaction was initiated and detected with ABI Prism 7500 RT PCR instrument. The relative level of mRNA was quantified with the 2^-△△Ct^ method. The primers were as follows: U6-forward: 5′-GCTTCGGCAGCACATATACTAAAAT-3′ and U6-reverse: 5′-CGCTTCACGAATTTGCGTGTCAT-3′; miR-3613-5p-forward: 5′-CTTGTTTTTTTTTTCATGTTGT-3′ and miR-3613-5p-reverse: 5′-AGTCTCAGGGTCCGAGGTATTC-3′; PTEN-forward: 5′-TGGATTCGACTTAGACTTGACCT-3′ and PTEN-reverse: 5′-GGTGGGTTATGGTCTTCAAAAGG-3′; and GAPDH-forward: 5′-GTCTCCTCTGACTTCAACAGCG-3′ and GAPDH-reverse: ACCACCCTGTTGCTGTAGCCAA.

For generation of PTEN knockdown cell line, the primers used to generate into pLKO.1-puro vector were as follows: sh-NC-sense strand: 5′-ACTGCCCTGATGCTAGCTAGCACCGGT-3′ and sh-NC-antisense strand: 5′-GCUCGATCCTGCTAGATCUUCGCUAC-3′; sh-PTEN-sense strand: 5′-GACAAAGCCAACCGATACTTT-3′; and sh-PTEN-antisense strand: 5′-AAAGTATCGGTTGGCTTTGTC-3′.

### 2.6. Exosome Isolation

Exosomes were isolated and purified with an ExoQuick precipitation kit (System Biosciences, LLC, Palo Alto, CA). Briefly, cell culture medium was collected and centrifuged at 3000 × g for 15 min. Supernatant was collected and mixed with ExoQuick precipitation solution. The mixture was incubated at 4°C for 30 min and centrifuged at 1500 × g for 30 min. The supernatant was carefully removed and resuspended in 100 *μ*l PBS.

### 2.7. Flow Cytometry

Cells were collected and washed with prechilled PBS. Cells were incubated with Annexin V-PE/7-AAD and propidium iodide (PI) for 10 min at room temperature in accordance with the manufacturer's instruction. Cell apoptosis was detected with a flow cytometer.

### 2.8. Soft Agar Colony Formation Assay

The base layer was prepared with 5 ml rich medium supplemented with 0.75% agar. The top layer was prepared with 3 ml rich medium supplemented with 0.36% agar at a concentration of 3 × 10^4^ cells/ml, incubated at 37°C for 3 weeks, and stained with 0.04% crystal violet in PBS and photographed with a scanner.

### 2.9. Dual-Luciferase Activity Assay

Cells were harvested and washed with PBS by centrifugation at 600 × g for 5 min. Cells were resuspended in reporter lysis buffer and kept on ice for 20 min. After a centrifugation at maximum speed for 10 min, the supernatant was collected. 20 *μ*L supernatant and 100 *μ*L luciferase assay reagent were mixed together. A luminometer was used to detect the fluorescence.

### 2.10. Cell Transfection

NC mimic, miR-3613-5p-mimic, and miR-3613-5p inhibitor were synthesized by GenePharma. Cells were transfected with a polyethylenimine- (PEI-) mediated method. Briefly, DNA was mixed with PEI at a ratio of 1 : 3 and diluted with free DMEM medium, followed by incubation at room temperature for 15 min. The mixture was added to the cell culture rich medium.

### 2.11. Western Blotting

Cells were harvested and washed with PBS for three times by centrifugation at 600 × g for 5 min. Cells were lysed in RIPA lysis buffer supplemented with protease and phosphatase inhibitors. Proteins were subjected to SDS-PAGE electrophoresis and transferred to PVDF membranes. The membranes were blocked with 5% (w/v) dry milk and then incubated with corresponding primary antibodies at 4°C overnight. The membranes were washed with 1× TBST for three times and then incubated with an HRP conjugated secondary antibody at room temperature for 1 h. After the membranes were washed with 1× TBST for three times, an enhanced chemiluminescence was used to visualize the blots. The primary antibodies were supplied by Abcam (Cambridge, UK). The information of antibodies was as follows: TSG101 (ab30871), CD63 (ab134045), PTEN (ab32199), and GAPDH (ab8245). All the antibodies were diluted in TBST at 1 : 1000.

### 2.12. Statistical Analysis

Data shown are as mean ± SD. Statistical significance was evaluated by GraphPad Prism software. Student's *t*-test or two-way ANOVA was used for statistical analysis. *p* < 0.05 was considered as statistically significant.

## 3. Results

### 3.1. miR-3613-5p Is Upregulated in Drug-Resistant Tissue and Breast Cancer Cells

**Figure 1:**
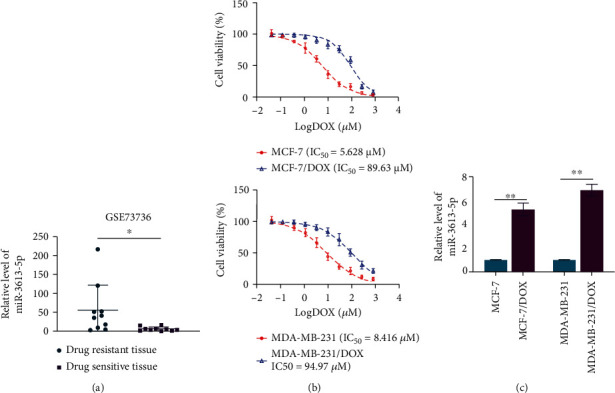
miR-3613-5p is upregulated in drug-resistant tissue and breast cancer cells. (a) The expression level of miR-3613-5p in GEO chip (GSE73736) of drug-sensitive tissue and drug-resistant tissue. ^∗^*p* < 0.05, *n* = 10. (b) Cell viability of doxorubicin nonresistant and resistant breast cancer cell lines (MCF-7 and MDA-MB-231, MCF-7/DOX, and MDA-MB-231/DOX, respectively) after different doses of doxorubicin treatments was assessed with CCK8. (c) qRT-PCR was performed to assess the relative levels of miR-3613-5p in MCF-7, MCF-7/DOX, MDA-MB-231, and MDA-MB-231/DOX. ^∗∗^*p* < 0.01. Data are mean ± S.D. of 3 independent experiments.

In drug-resistant tissue, the expression of miR-3613-5p was upregulated compared with drug-sensitive tissue (Figure 1(a)). Exosome-mediated transfer of long noncoding RNA H19 was used to generate resistant breast cancer cells to doxorubicin [14, 15]. Breast cancer cells became significantly resistant to the cytotoxicity of doxorubicin (Figure 1(b)). In breast cancer cells resistant to doxorubicin, the expression of miR-3613-5p was significantly increased (Figure 1(c)). These data demonstrated miR-3613-5p was upregulated in drug-resistant tissue and in breast cancer cells resistant to doxorubicin.

### 3.2. Expression of miR-3613-5p Is Upregulated in Exosomes of Doxorubicin-Resistant Breast Cancer Cells

**Figure 2:**
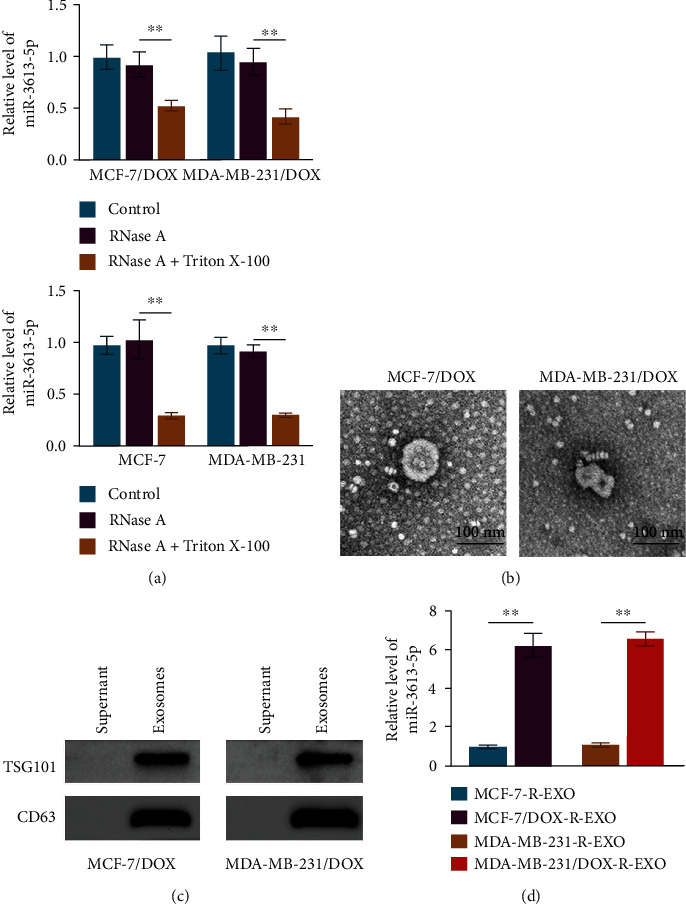
The expression of miR-3613-5p is upregulated in exosomes of doxorubicin-resistant breast cancer cells. (a) qRT-PCR was performed to assess the relative level of miR-3613-5p in MCF-7/DOX, MDA-MB-231/DOX, MCF-7, and MDA-MB-231 after the treatments of RNase A and Triton X-100. ^∗∗^*p* < 0.01. Data are mean ± S.D. of 3 independent experiments. (b) Exosomes were isolated, and the structure was observed by TEM. (c) Western blotting was used to detect the protein expression of TSG101 and CD63 in supernatant and exosomes of MCF-7/DOX and MDA-MB-231/DOX cells. (d) qRT-PCR was performed to assess the relative level of miR-3613-5p in the exosomes of MCF-7, MCF-7/DOX, MDA-MB-231, and MDA-MB-231/DOX. ^∗∗^*p* < 0.01. Data are mean ± S.D. of 3 independent experiments.

The addition of RNase A to the culture medium had no effect on the miR-3613-5p level, but the combined addition of Triton X-100 led to dramatical decrease in miR-3613-5p level (Figure 2(a)). This observation indicated that miR-3613-5p was surrounded by membranes but not directly released into the medium. Exosomes were isolated, the structure was observed by TEM, and the images showed that the particles were typical goblet-shaped vesicles with a double-membrane structure, approximately 100 nm in diameter (Figure 2(b)). Immunoblotting analysis of exosome markers TSG101 and CD63 confirmed the presence of exosome (Figure 2(c)). In the exosomes from doxorubicin-resistant breast cancer cells, the relative level of miR-3613-5p was significantly enhanced (Figure 2(d)). These observations demonstrated that miR-3613-5p level was upregulated in exosomes from doxorubicin-resistant breast cancer cells.

### 3.3. Exosome-Mediated miR-3613-5p Transfer Enhances the Resistance of Breast Cancer Cells to Doxorubicin

**Figure 3:**
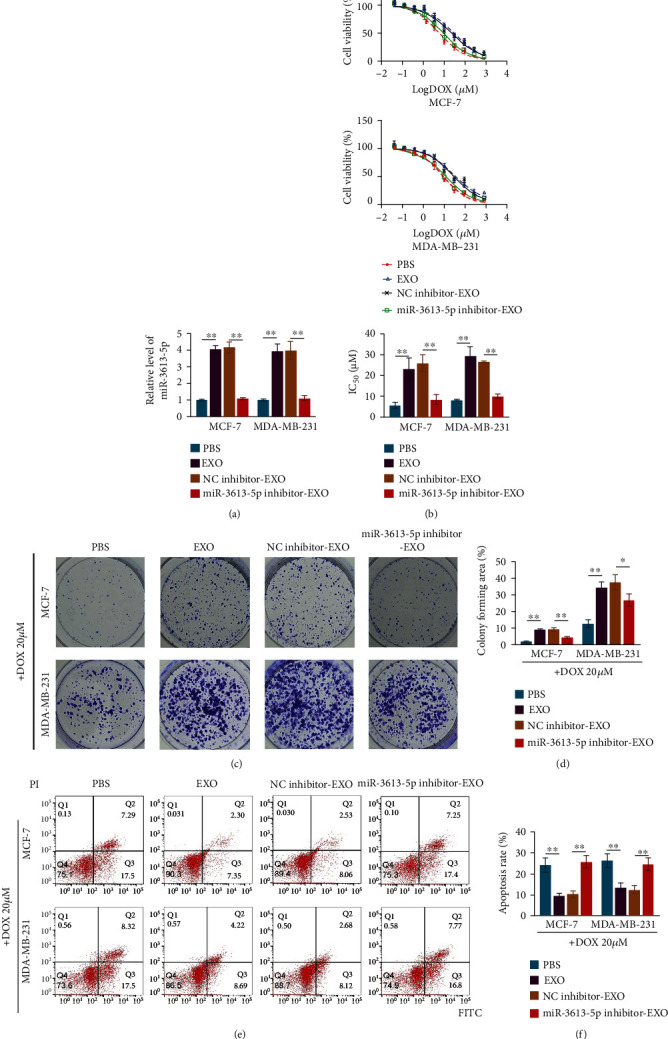
Exosome-mediated miR-3613-5p transfer enhances the resistance of breast cancer cells to doxorubicin. (a) qRT-PCR was used to assess the relative level of miR-3613-5p in MCF-7 and MDA-MB-231 cells after incubation with exosomes isolated from doxorubicin-resistant cells (EXO) and with the treatments of NC inhibitor (NC inhibitor-EXO) or miR-3613-5p inhibitor (miR-3613-5p inhibitor-EXO). ^∗∗^*p* < 0.01. Data are mean ± S.D. of 3 independent experiments. (b) CCK8 was used to assess cell viability of MCF-7 and MDA-MB-231 cells after the treatments of PBS, EXO, NC inhibitor-EXO, or miR-3613-5p inhibitor-EXO. (Upper and middle) Curve of cell viability after indicated treatments in MCF-7 and MDA-MB-231 cells. (Lower) Half maximal inhibitory concentration (IC_50_) values of doxorubicin (DOX) in MCF-7 and MDA-MB-231 cells. ^∗∗^*p* < 0.01. Data are mean ± S.D. of 3 independent experiments. (c) Crystal violet staining to detect colony formation of MCF-7 and MDA-MB-231 cells after the cells treated with 20 *μ*M DOX combined with treatments of PBS, EXO, NC inhibitor-EXO, or miR-3613-5p inhibitor-EXO. (d) Colony forming area (%) detected by crystal violet staining in (c). ^∗^*p* < 0.05, ^∗∗^*p* < 0.01. Data are mean ± S.D. of 3 independent experiments. (e, f) Flow cytometry was used to detect the cell apoptosis rate of MCF-7 and MDA-MB-231 cells after the cells treated with 20 *μ*M DOX combined with treatment of PBS, EXO, NC inhibitor-EXO, or miR-3613-5p inhibitor-EXO. ^∗∗^*p* < 0.01. Data are mean ± S.D. of 3 independent experiments.

Incubation with exosomes from doxorubicin-resistant breast cancer cells promoted the relative level of miR-3613-5p, and miR-3613-5p inhibitor led to a significantly decrease in miR-3613-5p level in exosomes from breast cancer cells (Figure 3(a)). Cell viability (Figure 3(b)), colony formation (Figures 3(c) and 3(d)), and flow cytometry (Figures 3(e) and 3(f)) analysis revealed that incubation with exosomes from doxorubicin-resistant breast cancer cells increased the cell resistance to doxorubicin, and miR-3613-5p inhibitor treatment sensitized cell death to doxorubicin (Figures 3(b)–3(f)). These results indicated that exosome mediated miR-3613-5p transfer and enhanced doxorubicin resistance in breast cancer cells.

### 3.4. miR-3613-5p Targets PTEN

**Figure 4:**
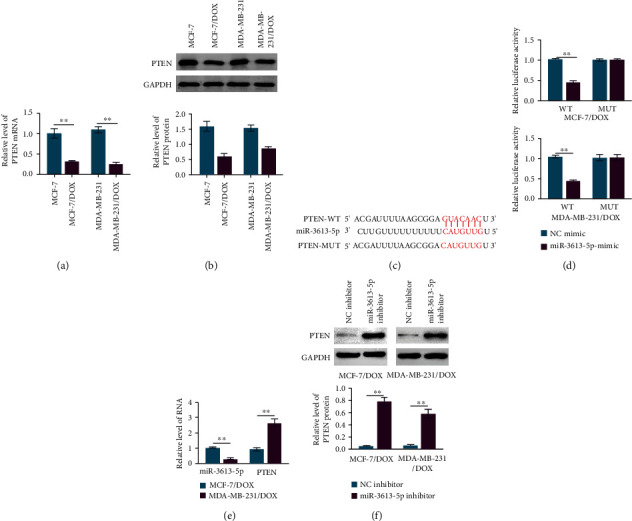
miR-3613-5p targets PTEN. (a) qRT-PCR was used to assess the relative level of PTEN in MCF-7, MCF-7/DOX, MDA-MB-231, and MDA-MB-231/DOX cells. ^∗∗^*p* < 0.01. Data are mean ± S.D. of 3 independent experiments. (b) Western blotting was used to detect the protein expression of PTEN in MCF-7, MCF-7/DOX, MDA-MB-231, and MDA-MB-231/DOX cells. ^∗∗^*p* < 0.01. Data are mean ± S.D. of 3 independent experiments. (c) Website TargetScan predicted the binding site of PTEN to miR-3613-5p. (d) Dual luciferase reporter assay was performed to detect the luciferase activity in wild-type (WT) and mutant (MUT) of MCF-7/DOX and MDA-MB-231/DOX cells after transfection with NC mimics and miR-3613-5p mimics. ^∗∗^*p* < 0.01. Data are mean ± S.D. of 3 independent experiments. (e) qRT-PCR was used to assess the relative levels of miR-3613-5p and PTEN in MCF-7/DOX and MDA-MB-231/DOX cells. ^∗∗^*p* < 0.01. Data are mean ± S.D. of 3 independent experiments. (f) Western blotting was used to detect the protein expression of PTEN in MCF-7/DOX and MDA-MB-231/DOX cells after the treatments of NC inhibitor and miR-3613-5p inhibitor. ^∗∗^*p* < 0.01. Data are mean ± S.D. of 3 independent experiments.

The molecular mechanism through which miR-3613-5p enhanced the resistance of breast cancer cells to doxorubicin was further explored. The relative mRNA and protein levels of PTEN were dramatically declined in doxorubicin-resistant breast cancer cells (Figures 4(a) and 4(b)). The website TargetScan predicted that miR-3613-5p could bind to PTEN (Figure 4(c)). The overexpression of miR-3613-5p induced the suppression of luciferase activity in wild-type, which was abolished in PTEN mutant, indicating that miR-3613-5p could interact with PTEN (Figure 4(d)). In MDA-MB-231 cells resistant to doxorubicin, the relative level of miR-3613-5p was much lower, while the relative level of PTEN was much higher than that in doxorubicin-resistant MCF-7 cells (Figure 4(e)). miR-3613-5p inhibitor strikingly enhanced the expression level of PTEN in doxorubicin-resistant breast cancer cells (Figure 4(f)). These data suggested that miR-3613-5p could target PTEN and regulate the expression of PTEN, which was involved in doxorubicin resistance of breast cancer cells.

### 3.5. Exosome-Mediated Transfer of miR-3613-5p Enhances the Resistance of Breast Cancer Cells to Doxorubicin by Targeting PTEN

**Figure 5:**
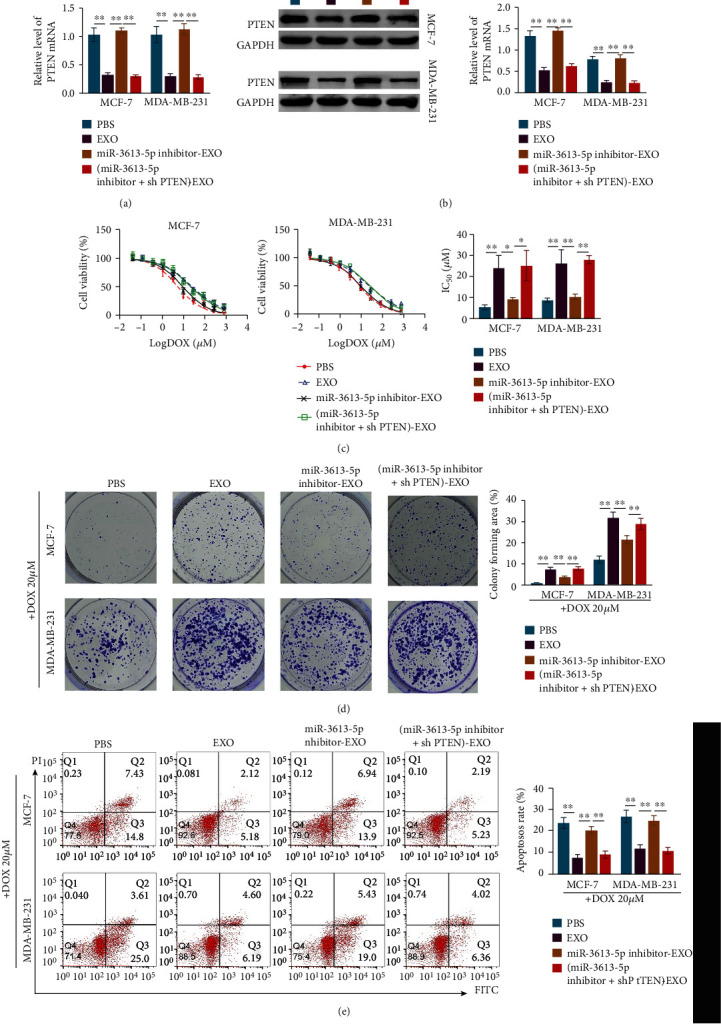
Exosome-mediated transfer of miR-3613-5p enhances the resistance of breast cancer cells to doxorubicin by targeting PTEN. (a) qRT-PCR was used to assess the relative level of PTEN in MCF-7 and MDA-MB-231 cells after incubation with PBS or exosomes isolated from doxorubicin resistant cells (EXO) and with the treatment of miR-3613-5p inhibitor (miR-3613-5p inhibitor-EXO) and knockdown of PTEN ((miR-3613-5p inhibitor+shPTEN)-EXO). ^∗∗^*p* < 0.01. Data are mean ± S.D. of 3 independent experiments. (b) Western blotting was used to detect the protein expression of PTEN in MCF-7 and MDA-MB-231 cells after treatments of PBS or EXO or miR-3613-5p inhibitor-EXO or (miR-3613-5p inhibitor+shPTEN)-EXO. ^∗∗^*p* < 0.01. Data are mean ± S.D. of 3 independent experiments. (c) CCK8 was used to assess cell viability of MCF-7 and MDA-MB-231 cells after the cells treated with PBS or EXO or miR-3613-5p inhibitor-EXO or (miR-3613-5p inhibitor+shPTEN)-EXO. (Left and middle) Curve of cell viability after indicated treatments in MCF-7 and MDA-MB-231 cells. (Right) IC_50_ values of doxorubicin (DOX) after indicated treatments in MCF-7 and MDA-MB-231 cells. ^∗^*p* < 0.05, ^∗∗^*p* < 0.01. Data are mean ± S.D. of 3 independent experiments. (d) Crystal violet staining to detect colony formation of MCF-7 and MDA-MB-231 cells after the cells treated with 20 *μ*M doxorubicin (DOX) combined with treatments of PBS or EXO or miR-3613-5p inhibitor-EXO or (miR-3613-5p inhibitor+shPTEN)-EXO. ^∗∗^*p* < 0.01. Data are mean ± S.D. of 3 independent experiments. (e) Flow cytometry was used to detect the cell apoptosis rate of MCF-7 and MDA-MB-231 cells after the cells treated with PBS or EXO or miR-3613-5p inhibitor-EXO or (miR-3613-5p inhibitor+shPTEN)-EXO. ^∗∗^*p* < 0.01. Data are mean ± S.D. of 3 independent experiments.

Incubation with exosomes from doxorubicin-resistant breast cancer cells or knockdown of PTEN led to the significant decrease in the PTEN expression, which was rescued by the treatment of miR-3613-5p inhibitor (Figures 5(a) and 5(b)). Incubation with exosomes from doxorubicin-resistant breast cancer cells or knockdown of PTEN enhanced the resistance to doxorubicin, which was prevented by the treatment of miR-3613-5p inhibitor (Figures 5(c)–5(e)). These data indicated that exosome-mediated transfer of miR-3613-5p enhanced the resistance of breast cancer cells to doxorubicin by inhibition of PTEN.

## 4. Discussion

Breast cancer is one of the most common malignancies with increasing incidence in women worldwide [1]. Doxorubicin is a well-accepted compound for breast cancer therapy, but patients easily develop doxorubicin resistance [3]. Therefore, it is urgent to further explore the molecular mechanisms of drug resistance and novel therapeutic strategy for breast cancer.

Exosomes participate in intercellular communication and mediate crosstalk between neighboring cells [7]. miRNAs are involved in many diseases and have been shown in the regulation of drug resistance [10, 11]. miRNAs are protected by bilateral membrane structures after incorporation into exosomes, which prevents the degradation of miRNAs and facilitates the intercellular communication [11]. miR-3613-5p is abnormally expressed and carcinogenic in a variety of tumors, including pancreatic cancer [16] and non-small-cell lung cancer [17]. miR-3613-5p can be present in exosomes [18]. However, whether exosome-mediated miR-3613-5p transfer can regulate the drug resistance and the molecular mechanism remains to be investigated.

According to GEO database analysis, miR-3613-5p is upregulated in drug-resistant breast cancer. It is predicted that miR-3613-5p can bind to PTEN, which is a well-known tumor suppressor gene that participates in tumor cell proliferation, cell apoptosis, invasion, migration, drug resistance, and many signaling pathways [19, 20]. It has been shown that inhibition of PTEN promotes cell proliferation of doxorubicin-resistant breast cancer cells and inhibits apoptosis, thus promoting drug resistance of breast cancer [21].

In this study, exosome-mediated transfer of long noncoding RNA H19 was used to generate doxorubicin-resistant breast cancer cells, and the expression of miR-3613-5p was significantly increased in these cells. It has been validated that miRNAs including miR-3613-5p was expressed in exosomes [18, 22], but the expression levels of miRNAs were significant differential [22]. In the exosomes from doxorubicin-resistant breast cancer cells, the relative level of miR-3613-5p was significantly enhanced. Incubation with exosomes from doxorubicin-resistant breast cancer cells promoted the relative level of miR-3613-5p and increased the resistance of breast cancer cells to doxorubicin. These results indicated that exosome mediated miR-3613-5p transfer and enhanced the resistance of breast cancer cells to doxorubicin. The molecular mechanism through which miR-3613-5p promoted drug resistance was then investigated. PTEN was known to be a key regulator of doxorubicin resistance in breast cancer [23]. miR-3613-5p could target PTEN and regulate the expression of PTEN, which was involved in doxorubicin resistance of breast cancer cells. Incubation with exosomes from doxorubicin-resistant breast cancer cells or knockdown of PTEN enhanced the resistance of breast cancer cells to doxorubicin, which was prevented by the treatment of miR-3613-5p inhibitor. These observations suggested that exosome-mediated transfer of miR-3613-5p enhanced the resistance of breast cancer cells to doxorubicin by inhibition of PTEN. This finding will provide a therapeutic target and strategy for breast cancer treatment.

## Data Availability

All data generated or analyzed during this study are included in this published article.
